# A composite endpoint for systemic sclerosis-associated interstitial lung disease: association with mortality in two clinical trial cohorts

**DOI:** 10.1186/s12931-025-03401-8

**Published:** 2025-11-28

**Authors:** Elizabeth R. Volkmann, Holly Wilhalme, Sam Good, Grace Hyun J. Kim, Jonathan Goldin, Michael D. Roth, Donald P. Tashkin

**Affiliations:** David Geffen School of Medicine, University of California, Los Angeles, 1000 Veteran Ave. Ste 32-59, Los Angeles, CA 90095 USA

**Keywords:** Systemic sclerosis, Interstitial lung disease, Patient outcome assessment, Randomized controlled trial

## Abstract

**Background:**

The forced vital capacity (FVC) is the most commonly used endpoint in registrational trials for systemic sclerosis-associated interstitial lung disease (SSc-ILD). However, the FVC has known methodological pitfalls, is affected by extra-pulmonary SSc manifestations and may not be clinically meaningful to patients. Combining individual outcomes into a composite endpoint is an attractive alternative for measuring treatment response and augmenting statistical efficiency in SSc-ILD trials.

**Methods:**

We previously developed a composite endpoint for SSc-ILD using data from the Scleroderma Lung Study (SLS) I (comparing cyclophosphamide versus placebo for SSc-ILD), which included physiological (FVC), radiological (quantitative extent of fibrosis in the zone of maximum involvement) and patient-reported outcomes (transitional dyspnea index and health assessment disability questionnaire), which demonstrated a more robust treatment effect of cyclophosphamide than the FVC alone. The purpose of this post-hoc analysis was to validate this composite endpoint in an external clinical trial cohort (SLS II [cyclophosphamide versus mycophenolate for SSc-ILD). A secondary goal was to determine whether the composite endpoint predicted long-term mortality in SLS I and II.

**Results:**

Seventy-two of the 142 randomized participants in SLS II had all composite endpoint components at 24 months and were included in this analysis. In both SLS I and II, the standardized effect size (Cohen’s d) was greater when the composite endpoint model was applied than when the FVC alone model was applied. In SLS I and II, the composite endpoint was a better predictor of long-term survival (HR 0.76 vs. 0.98 and 0.59 vs. 0.98, for the composite index vs. FVC Cox proportional hazards models in SLS I and II, respectively). Patients with high composite outcomes scores in both SLS I and II had significantly worse long-term survival than patients with low scores (*p* = 0.039 for log-rank test).

**Conclusion:**

The results of this post-hoc analysis provide further evidence that a composite endpoint comprised of physiological, radiological and patient-reported outcomes is a promising endpoint for SSc-ILD trials. Future validation studies are needed.

**Trial registration:**

SLS II: NCT00883129 (Date of registration April 17, 2009); SLS I: NCT00004563 (Date of registration: February 10, 2000).

**Supplementary Information:**

The online version contains supplementary material available at 10.1186/s12931-025-03401-8.

## Background

Systemic sclerosis-associated interstitial lung disease (SSc-ILD) is a chronic, progressive disease with a heterogenous course [1]. In clinical practice, disease progression of SSc-ILD is defined based on physiological (e.g., forced vital capacity [FVC], diffusing capacity for carbon monoxide [DLCO]) and radiological assessments in combination with respiratory symptoms and exercise tolerance evaluations [2]. By contrast, in clinical trials, the impact of interventions on disease progression has been measured by changes in FVC alone [3–6]. For a systemic disease with multiple extrapulmonary manifestations that affect FVC measurements (e.g., cutaneous sclerosis, myopathy, etc.), there are limitations of this singular approach.

Moreover, the FVC is subject to methodological pitfalls, including intrasubject variation based on diurnal variation, and technical factors (e.g., how well the mouthpiece fits in a patient with decreased oral aperture, which is common in SSc). Due to this variation, multiple FVC measurements are required over the course of one or more years to establish clear disease progression trends, and relatively large groups of patients are needed to discern treatment effects on FVC course. Furthermore, including individuals with a slower rate of decline in FVC in the primary analysis may obscure observable treatment effects that occur in rapidly progressive SSc-ILD subgroups [7]. In addition, a major focus on this one dimension of SSc-ILD may misrepresent meaningful changes in patient-centered outcomes, including improvements in symptoms and quality of life not reflected in changes in FVC.

Composite endpoints represent a promising solution to advance clinical development in ILD [8]. In addition to including outcomes that may be more meaningful to the patient (e.g., respiratory symptoms and quality of life), composite endpoints may also serve to reduce the number patients needed in a clinical trial to achieve adequate statistical power to detect treatment effects [9]. In the current SSc-ILD trial landscape, which includes multiple competing trials with similar entry criteria, trials with smaller sample sizes are desirable. With a smaller target sample size, the length of the overall study may also be curtailed when a composite endpoint is used due to shorter accrual periods, which may also potentially lowering overall sponsor costs. While there is a risk of diluting treatment effects when individual components of the composite endpoint with no observable treatment effects are combined with those with stronger trials effects [9], most trials report results of the individual components separately as key secondary outcomes.

The primary objective of the present post-hoc analysis was to validate a composite endpoint that we previously developed from the Scleroderma Lung Study (SLS) I clinical cohort [10] in an external cohort of patients who participated in the SLS II. Both SLS I (comparing cyclophosphamide [CYC] to placebo [3]) and II (comparing CYC and mycophenolate mofetil [MMF] [4]) were randomized controlled trials (RCTs) for SSc-ILD. A secondary objective was to determine whether the composite endpoint predicted long-term mortality in SLS I and II. We hypothesized that the composite endpoint would perform similarly in SLS II as in SLS I and would predict long-term mortality in both cohorts.

## Methods

### Patient population

We used patient data from SLS II (NCT00883129) [4] to test the performance of the composite endpoint [10] developed based on the SLS I cohort (NCT00004563) [3] as these two multi-institutional, double-blind, RCTs for SSc-ILD had similar entry criteria and outcome measurements. The common inclusion criteria included the following: (1) SSc based on the 1980 ACR criteria; age ≥ 18 years; (2) disease duration ≤ 7 years from the onset of the first non-Raynaud symptom of SSc; (3) forced vital capacity (FVC) 45–85% predicted; (4) hemoglobin-adjusted single-breath diffusing capacity for carbon monoxide (DLCO-Hb) ≥ 40% predicted (or 30–39% predicted in the absence of evidence of clinically significant pulmonary hypertension); and (5) evidence of any ground glass opacity, defined as any hazy parenchymal opacity in the presence or absence of reticular opacity or architectural distortion on high-resolution computed tomography (HRCT) of the chest. All participants of SLS I and II provided their informed consent to participate in these studies in accordance with the Declaration of Helsink.

### Composite endpoint measurements

The individual components of the composite endpoint derived from the SLS I cohort included the FVC%-predicted, quantitative lung fibrosis score in the zone of maximum involvement (QLF-ZM), Transitional Dyspnea Index (TDI) and the Health Assessment Questionnaire-Disability Index (HAQ-DI) [10]. Each of these measures are commonly used in SSc-ILD trials and are individually known to have an impact on patients with SSc-ILD. Briefly, a principal component analysis (PCA) was used to reveal how these different variables change in relation to each other through transforming correlated original variables into a new set of uncorrelated variables using a covariance matrix [10]. The first principal component was calculated from the analysis and then was used as the outcome in a linear regression model. The regression model included the group indicator (CYC vs. placebo for SLS I and CYC vs. MMF for SLS II) as the primary predictor, while adjusting for the first principal component calculated using baseline data. The model also considered a possible interaction effect between group and the baseline principal component. The weights for each component reflect the relative importance of each component in capturing the overall variability of the data. For example, higher explained variance ratios suggest that a component has a more significant impact on the overall variance and should be considered more important when determining the weights for the index components.

A notable difference between the SLS I and II study design was the timing of HRCT assessment (i.e., 12 months in SLS I and at 24 months in SLS II). In order to assess the performance of the complete composite endpoint, including the radiological assessment measure, the composite endpoint was evaluated at 24 months in SLS II.

### Validation process

To validate the composite endpoint in SLS II, we performed four distinct validation procedures. First, we compared the distribution of composite endpoint scores between the two cohorts to determine whether the composite endpoint generated outputs that were comparable between the two cohorts and thereby assess its generalizability.

Second, we evaluated the stability of the weighting structure for the composite endpoint in SLS I and II by recalculating factor loadings for the individual composite endpoint components and thereby determined the robustness of the weights. Since the original weighting structure was data driven (i.e. based on a PCA]), we wanted to ensure that we could generate similar factor loadings for the individual composite endpoint components using SLS II data using the same statistical approach as in SLS I.

Third, we evaluated the composite endpoint’s ability to discriminate between treatment arms. In contrast to SLS I, in which a treatment effect favoring CYC over placebo was observed, SLS II did not have a placebo arm. Instead, SLS II compared two active study medications (CYC vs. MMF), and no differences were observed in any of the composite endpoint components in SLS II. While we did not anticipate that the composite endpoint would demonstrate a differential treatment effect between MMF and CYC, we proceeded to compare the standardized effect sizes based on Cohen’s d between the composite endpoint and the change in FVC%-predicted at 24 months in SLS II. We performed a sample size calculation for a two-sample t-test with 80% power and alpha of 0.05 for the composite outcome and compared this with the same sample size calculation using the change in FVC% predicted.

The fourth and final step of validation was to determine whether the composite endpoint predicted long-term survival in SLS II, as well as in SLS I. Transplant-free survival was captured up to 12 years and 8 years from the time that the first patient was randomized in SLS I and II, respectively, as previously described [11]. Time zero for the survival analysis was the time the patient entered the trial. To determine whether the composite outcome predicted long-term survival, patients were dichotomized based on the median composite outcome score (calculated at 12 months in SLS I and 24 months in SLS II). The Kaplan-Meier method was used to compare transplant-free survival rates between the two subgroups. We also performed a Cox proportional hazards model analysis using the composite outcome score (measured continuously) as a covariate and compared the hazards ratio of the composite outcome score covariate to the hazards ratio of the change in FVC covariate.

## Results

### Patient characteristics

Of the 142 participants of SLS II, 72 participants (*N* = 35 CYC, *N* = 37 MMF) had all composite endpoint measurements performed at 24 months and were included in this post-hoc analysis. The mean FVC%-predicted for all 72 participants was 67.0 (SD 8.95), and the mean quantitative extent of fibrosis in the whole lung (QLF-WL) was 8.3 (SD 7.32)%, while the QLF-ZM was 23 (SD 21.75)%. The baseline characteristics of the 72 participants included in the present analysis were similar to the baseline characteristics of the 70 participants who did not have all composite endpoint measurements performed at 24 months, as well as the overall trial cohort (Table 1). The number of assessments missing for the individual components of the composite endpoint are summarized in Supplementary Table 1.

**Table 1:** Baseline characteristics of SLS II participants who were included (*N* = 72) and not included (*N* = 70) in the present post-hoc analyses. The baseline characteristics of the entire SLS II cohort are provided as a reference

	Included in Analysis	Not Included in Analysis	Total
Age			
N	72	70	142
Mean (SD)	50.6 (8.44)	54.0 (10.68)	52.3 (9.73)
Median	51.0	53.5	52.5
Range	29.0, 66.0	28.0, 79.0	28.0, 79.0
Sex, n (%)			
N	72	70	142
Male	19 (26.4%)	18 (25.7%)	37 (26.1%)
Female	53 (73.6%)	52 (74.3%)	105 (73.9%)
Disease Duration (years)			
N	72	67	139
Mean (SD)	2.7 (1.79)	2.5 (1.75)	2.6 (1.77)
Median	2.3	1.8	1.9
Range	0.3, 7.1	0.3, 6.9	0.3, 7.1
Diffuse, n (%)			
N	72	70	142
Limited	32 (44.4%)	27 (38.6%)	59 (41.5%)
Diffuse	40 (55.6%)	43 (61.4%)	83 (58.5%)
Treatment Arm, n (%)			
N	72	70	142
CYC	35 (48.6%)	38 (54.3%)	73 (51.4%)
MMF	37 (51.4%)	32 (45.7%)	69 (48.6%)
BDI			
N	72	62	134
Mean (SD)	7.4 (2.35)	7.0 (2.02)	7.2 (2.20)
Median	7.0	7.0	7.0
Range	0.0, 12.0	1.0, 11.0	0.0, 12.0
HAQ-DI			
N	72	70	142
Mean (SD)	0.6 (0.63)	0.8 (0.71)	0.7 (0.68)
Median	0.4	0.8	0.6
Range	0.0, 2.3	0.0, 3.0	0.0, 3.0
mRSS			
N	72	70	142
Mean (SD)	14.5 (9.69)	14.9 (11.35)	14.7 (10.51)
Median	13.0	12.0	13.0
Range	2.0, 41.0	1.0, 46.0	1.0, 46.0
FVC%-predicted			
N	72	70	142
Mean (SD)	67.0 (8.95)	66.0 (9.35)	66.5 (9.13)
Median	67.9	65.6	67.4
Range	44.8, 82.9	47.1, 85.8	44.8, 85.8
DLCO %-predicted			
N	72	70	142
Mean (SD)	54.1 (12.63)	53.9 (12.78)	54.0 (12.66)
Median	51.3	52.0	51.5
Range	31.9, 86.7	32.5, 88.8	31.9, 88.8
QILD-WL			
N	72	65	137
Mean (SD)	28.3 (13.08)	30.8 (14.91)	29.5 (13.99)
Median	25.4	29.5	27.8
Range	8.8, 68.0	6.2, 66.3	6.2, 68.0
QILD-ZM			
N	72	65	137
Mean (SD)	50.9 (20.71)	51.5 (20.06)	51.2 (20.33)
Median	48.3	55.1	53.5
Range	15.5, 93.4	5.0, 92.5	5.0, 93.4
QLF-WL			
N	72	65	137
Mean (SD)	8.3 (7.32)	8.9 (6.51)	8.6 (6.93)
Median	6.4	8.4	7.3
Range	0.5, 32.0	0.3, 28.3	0.3, 32.0
QLF-ZM			
N	72	65	137
Mean (SD)	23.0 (21.75)	22.6 (17.15)	22.8 (19.63)
Median	17.4	20.6	18.1
Range	0.9, 84.5	0.4, 85.0	0.4, 85.0

*Abbreviations:* *FVC* Forced vital capacity, *DLCO* Diffusing capacity for carbon monoxide, *CYC* Cyclophosphamide, *MMF* Mycophenolate mofetil, *QLF-ZM* Quantitative lung fibrosis score in the zone of maximum involvement, *QLF-WL* Quantitative lung fibrosis score in the whole lung, *TDI* Transitional dyspnea index, *HAQ-DI* Health assessment questionnaire-disability index

### Validation of composite endpoint

**Fig. 1:**
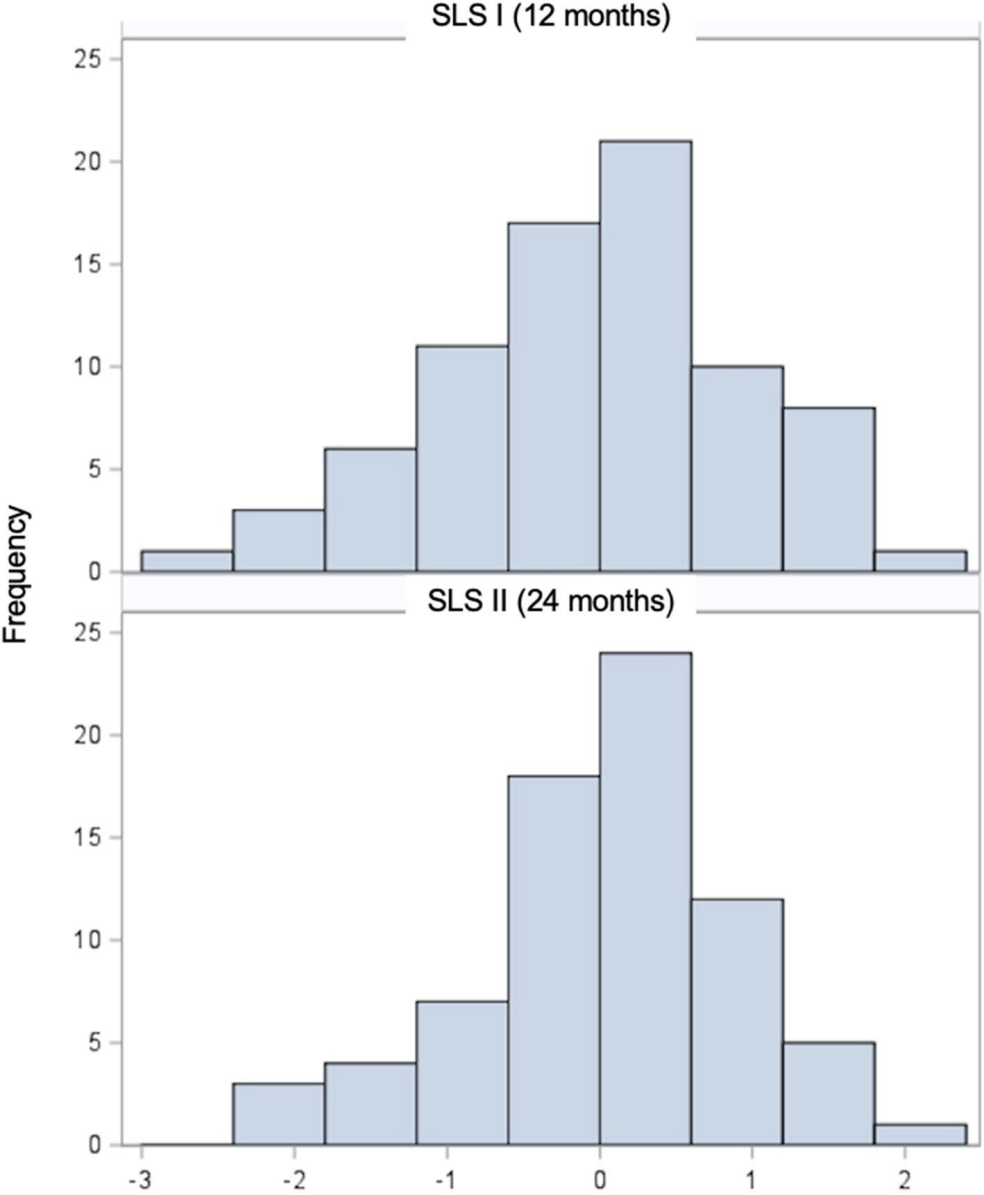
Histogram of composite index distributions in SLS I (top, composite endpoint measured at 12 months) and SLS II (bottom, composite endpoint measured at 24 months)

The composite endpoint score distributions for SLS I and SLS II were highly similar with a range of −2.8 to 1.9 and − 2.1 to 1.8, in SLS I and II, respectively (Fig. 1). In SLS II, the composite endpoint had a mean of 0.06 (SD 0.86), indicating slightly reduced variability compared to SLS I (standard normal). The mean composite endpoint score was also similar between treatment arms in SLS I (CYC = 0.27 [SD 0.87]; Placebo − 0.27 [SD 1.06]) and in SLS II (CYC = 0.02 [SD 0.92]); MMF = 0.09 [SD 0.81]).

In terms of the individual components of the composite endpoint, all components (e.g., TDI, FVC, HAQ-DI) improved over the course of 24 months in SLS II, with the exception of the QLF-ZM, which slightly increased, i.e., worsened (Table 2).

**Table 2:** Mean (SD) change from baseline in the individual components of the composite endpoint in SLS II

Variable	Mean (SD) change from baseline to 24 months		
	Overall	CYC	MMF
TDI^*^	2.11 (3.92)	2.17 (3.95)	2.05 (3.95)
QLF-ZM	1.82 (9.85)	1.08 (8.54)	2.56 (11.04)
FVC	4.00 (7.65)	3.48 (8.72)	4.41 (6.55)
HAQ - DI^†^	−0.04 (0.55)	−0.06 (0.41)	−0.03 (0.67)

*Abbreviations*: *FVC* Forced vital capacity, *CYC* Cyclophosphamide, *MMF* Mycophenolate mofetil, *QLF-ZM* Quantitative lung fibrosis score in the zone of maximum involvement, *TDI* Transitional dyspnea index, *HAQ-DI* Health assessment questionnaire-disability index

^*^Increase in TDI indicates improvement

^†^Decrease in HAQ-DI indicates improvement

The underlying weighting structure across SLS I and II was also stable (Table 3). As demonstrated in Table 3, when new factor loadings were calculated in the SLS II cohort using the same methodology as in SLS I [10], the weights for the individual components of the composite endpoint were fairly similar. Of all of the components, the recalculated HAQ-DI weight was the only component that was somewhat different in the SLS II cohort compared with the SLS I cohort (more influential). As mentioned in the methods, we used the original weighting structure from SLS I to validate the composite endpoint in the SLS II cohort; however, the purpose of comparing factor loadings of the individual components of the composite endpoint between the two cohorts was to assess generalizability.

**Table 3:** Factor loadings of the individual components of the composite endpoint in SLS II compared with SLS I. The underlying weighting structure across the two datasets is stable demonstrating that the composite endpoint is generalizable and captures the same information

Component	SLS I Weights	SLS II Weights
TDI	0.38	0.45
QLF - ZM	−0.39	−0.28
FVC	0.41	0.43
HAQ-DI	−0.14	−0.36

*Abbreviations:* *FVC* Forced vital capacity, *QLF-ZM* Quantitative lung fibrosis score in the zone of maximum involvement, *TDI* Transitional dyspnea index, *HAQ-DI* Health assessment questionnaire-disability index

## Comparing treatment effects of composite endpoint and FVC

To compare treatment effects of the composite endpoint model with the change in FVC model, we calculated the Cohen’s d (standardized effect size) for each model (Table 4). In SLS I, the Cohen’s d of the composite endpoint model was higher than that of the model that used FVC as the endpoint (0.85 vs. 0.36). In SLS II, the Cohen’s d of the composite endpoint model was slightly higher than that of the model that used FVC as the endpoint (0.094 vs. −0.071), acknowledging that no treatment arm effects were observed in any of the composite endpoint components in SLS II, including the FVC.

**Table 4:** Comparison of the treatment effects between the composite endpoint model and the FVC model in SLS I and II

SLS I		
	Composite Endpoint Model	FVC Model
Treatment Effect (CYC vs. placebo)	0.696 (Std Error 0.193), *p* = 0.0006	3.019 (Std Error 1.885), *p* = 0.1133
Cohen’s d (Standardized Effect Size)	0.848	0.359
Sample Size Needed to Detect Treatment Effect (per group)	23	123

SLS II		
Treatment Effect	0.061 (Std Error 0.153), *p* =	−0.184 (Std Error 1.594),
(CYC vs. MMF)^*^	0.692	p = 0.909
Cohen’s d (Standardized Effect Size)	0.094	−0.071

*Abbreviations*: *FVC* Forced vital capacity, *CYC* Cyclophosphamide, *MMF* Mycophenolate mofetil

^*^In SLS II, there was no significant difference in the FVC endpoint, nor any of the other efficacy endpoints included in the composite endpoint, between patients randomized to CYC vs. MMF; therefore, a sample size calculation was not performed

### Sample size calculation

Using the composite endpoint model drastically reduced the sample size needed to detect a significant treatment effect compared with the FVC model in the SLS I cohort. For a two-sample t-test with 80% power and an alpha of 0.05, the number of patients needed for a study using the composite endpoint was 23 compared with 123 for a study using the change in FVC as the endpoint. We did not perform a sample size calculation using the SLS II dataset since SLS II lacked a placebo arm, and there were no observable differences in study outcomes between the two study arms [4].

### Long-term mortality

**Fig. 2:**
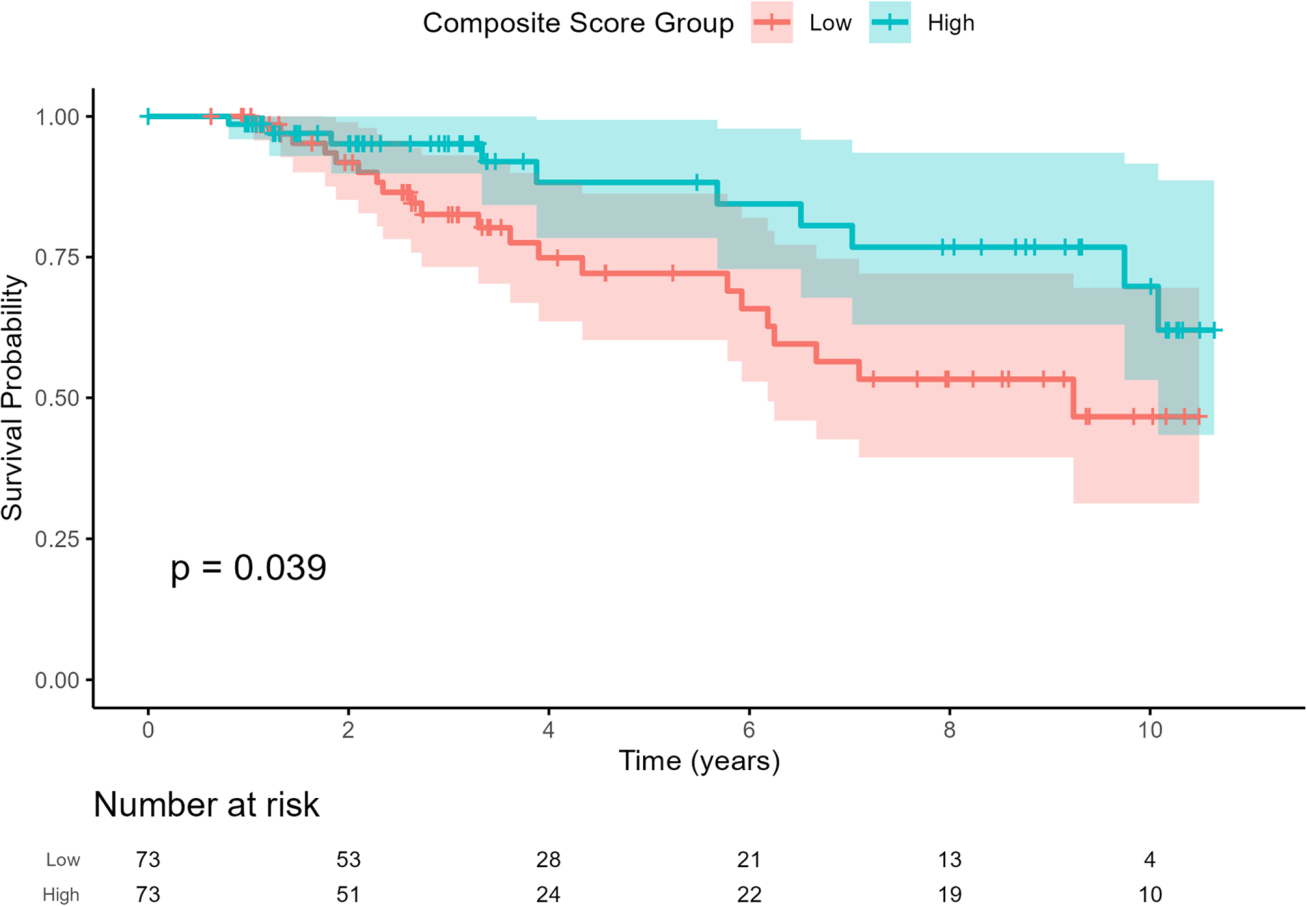
Kaplan Meier curve comparing transplant-free survival for SLS I and II patients with high and low composite endpoint scores (based median)

Of the participants included in the composite endpoint analysis in SLS I and II, 27 and 4 patients, respectively, died or had a lung transplant during the long-term follow up study. Because the number of patients who died or had lung transplant in the SLS II cohort was so small, we combined both cohorts to perform the Kaplan-Meier analysis. Patients with high composite outcome scores in the combined cohort (based on the median score) had an increased risk of death or lung transplantation compared with patients with low composite outcome scores, and this difference was statistically significant (*p* = 0.039 for log-rank test) (Fig. 2). The composite outcome score that best predicted survival was − 0.08 (c-statistic 0.63).

For the Cox proportional hazards model analysis in the SLS I cohort, the hazards ratio of death or lung transplantation was lower when the composite endpoint was used compared with the change in FVC, indicating that the composite endpoint was a better predictor of mortality compared with the change in the FVC (Table 5). In SLS II, the hazards ratio of death or lung transplantation was also lower when the composite endpoint was used compared with the change in FVC. Neither the composite endpoint, nor the change in FVC, was significantly associated with long-term mortality in the Cox models; however, these post-hoc analyses were not properly powered to detect statistical significance, particularly with the paucity of events.

**Table 5:** Cox proportional hazards model analysis for predicting long-term mortality in SLS I and II: comparison of using the composite endpoint vs. the change in FVC. Follow up time for patients in SLS I and II was up to 12 and 8 years, respectively, from the time the first patient was randomized in each study

Cohort	*N* = Deaths/Total	Hazards Ratio (CI)	*P*-value	C-Statistic
SLS I				
Composite index (12 months)	22/77	0.763 (0.529–1.099)	0.1462	0.61
Change in FVC (12 months)	22/77	0.982 (0.956–1.008)	0.1642	0.58
SLS II				
Composite index (24 months)	4/69	0.589 (0.190–1.830)	0.3604	0.71
Change in FVC (24 months)	4/69	0.980 (0.897–1.071)	0.6575	0.59

*Abbreviations:* *FVC* Forced vital capacity

## Discussion

Composite endpoints are commonly used as the primary measure of efficacy in cardiovascular disease and oncology clinical trials to assess overall treatment effects and increase the efficiency of trials [12]. While composite endpoints have been used in some pulmonary disease clinical trials (chronic obstructive pulmonary disease and asthma [13–15]), they have not been used in ILD clinical trials, despite growing concerns about the limitations of using the FVC alone as the primary endpoint [8]. In the present post-hoc analysis, we demonstrated that a composite endpoint comprised of physiological, radiological and patient-reported outcomes predicted future long-term mortality in two independent clinical trial cohorts. We also confirmed that the components of this composite endpoint behaved similarly in the SLS I and II cohorts. In addition, we discovered that the standardized effect size was larger when the composite endpoint was used compared with the FVC alone. Collectively, these data suggest that in addition to providing a more complete assessment of treatment impact on patients, the present composite endpoint is robust, generalizable and its use would reduce the sample size needed to detect a significant treatment effect compared with using the FVC as an endpoint.

While the present composite endpoint includes the FVC, it also includes a radiological component that approximates the burden of fibrosis, representing a unique aspect of disease progression not fully captured by pulmonary function testing. Changes in the quantitative extent of radiological fibrosis and ILD are independently associated with long-term mortality in SSc-ILD [16] and with patient-reported dyspnea and cough [17].

This composite endpoint also includes two patient-reported outcomes measures: the TDI and HAQ-DI. The former patient-reported outcome measures dyspnea, while the latter measures functional capacity; both are considered valid outcomes measures for SSc and have established minimum clinically important difference [MCID] thresholds for improvement [17]. While the HAQ-DI captures aspects of functionality (e.g., walking, climbing stairs), which directly relate to lung disease severity, it also addresses other aspects of functionality (e.g., eating, bathing, dressing) more related to extra-pulmonary manifestations of SSc (e.g., hand contractures, skin tightening). Interestingly, in SLS II, HAQ-DI scores correlated significantly (*r* = 0.41) with mRSS scores, but not with any measure of ILD severity (e.g., FVC, DLCO, quantitative imaging scores scores) [17]. Thus, the present composite endpoint may provide insight into treatment impact beyond the lungs in SSc. Moreover, since the HAQ-DI is a valid outcome measure for other rheumatic diseases (e.g., rheumatoid arthritis [18]), this composite endpoint may have relevance to clinical trials involving patients with ILD due to other underlying systemic autoimmune rheumatic diseases.

Unlike in the context of a placebo-controlled trial, we did not anticipate that the composite endpoint would demonstrate a significant treatment effect in SLS II, since none of the individual components were significantly different between the two treatment arms. We were able to demonstrate, however, that the standardized effect size was greater when the composite endpoint model was used compared to the FVC alone model in both cohorts. In addition, when we re-derived the factor loadings for the individual components of the composite endpoint using the SLS II cohort, the weighting structure remained similar. Finally, the composite endpoint score distributions for SLS I and II were nearly identical, increasing our confidence in the generalizability of this endpoint.

A major strength of this validation study is our access to long-term mortality data from both cohorts. ILD clinical trials rarely evaluate mortality beyond one to two years. For a disease such as SSc-ILD, which generally progresses at a slower rate than idiopathic pulmonary fibrosis, deaths occur infrequently during this time frame making it difficult to understand how a particular treatment affects survival. The fact that our composite endpoint predicted transplant-free survival in both cohorts suggests that it may be a suitable surrogate for mortality in other SSc-ILD populations.

Our study is not without limitations. In both SLS I and II, the composite endpoint was evaluated in a subgroup of patients from both studies who had all outcome components of the composite endpoint measured at the conclusion of the study, raising the possibility of selection bias. While the baseline characteristics of the patients included and not included in the composite endpoint analyses were reassuringly similar in both SLS I and II, we do not know whether excluding the patients who were missing from both analyses affected the performance of the composite outcome.

Another limitation of the study is that the radiological component of the composite endpoint was measured at 12 months in SLS I and 24 months in SLS II. We hypothesize that the greatest change in QLF-ZM would likely occur in the first 12 months, as we observed the greatest change in FVC over this time period in both SLS I and II [3, 4]. This difference in assessment testing may explain why the factor loading for the QLF-ZM component of the endpoint was slightly lower when we recalculated it based on the SLS II data (Table 2). However, this weakness could also represent a strength as we demonstrate that the composite endpoint may perform similarly whether it is applied to a one- or two-year clinical trial.

A third limitation is that we could not perform a sample size calculation using the SLS II dataset since there were no between treatment differences in the composite endpoint components. This would have provided important information for the design of current SSc-ILD trials, which allow for background use of MMF.

## Conclusion

To summarize, the present post-hoc analysis demonstrates the generalizability of a novel composite endpoint for SSc-ILD clinical trials. The use of this endpoint in future SSc-ILD clinical trials may not only improve trial feasibility (e.g., lower patient recruitment and enrollment burden, trial length, and cost), but it may also provide information on treatment effectiveness in ways that are more meaningful to both payers and patients. Future studies are needed to test this composite endpoint in other SSc-ILD trial cohorts.

## Supplementary Information

Supplementary Material 1.

## Data Availability

The datasets analyzed for the current study are available from the corresponding author on reasonable request.

## Abbreviations

SSc: Systemic sclerosis
ILD: Interstitial lung disease
FVC: Forced vital capacity
DLCO: Diffusing capacity for carbon monoxide
SLS: Scleroderma Lung Study
CYC: Cyclophosphamide
MMF: Mycophenolate mofetil
RCTs: Randomized controlled trials
HRCT: High-resolution computed tomography
QLF-ZM: Quantitative lung fibrosis score in the zone of maximum involvement
QLF-WL: Quantitative lung fibrosis score in the whole lung
TDI: Transitional dyspnea index
HAQ-DI: Health assessment questionnaire-disability index
SD: Standard deviation
