# Supplementary material for: A composite endpoint for systemic sclerosis-associated interstitial lung disease: association with mortality in two clinical trial cohorts
Source: Respir Res. 2025 Nov 28;26:337. doi: 10.1186/s12931-025-03401-8 (PMC12661867; doi:10.1186/s12931-025-03401-8)
Supplement: Supplementary file 1 — Supplementary Material 1. [file 12931_2025_3401_MOESM1_ESM.docx]

**Supplementary Table 1. Number of missing assessments for the individual components of the composite outcome at 24-months.**

|  |  | |  |
| --- | --- | --- | --- |
|  | **CYC (N=73)** | **MMF (N=69)** | **Total (N=142)** |
| **FVC% Predicted (24 months)** |  |  |  |
| N (Missing) | 51 (22) | 53 (16) | 104 (38) |
| **QLF-ZM (24 months)** |  |  |  |
| N (Missing) | 47 (26) | 51 (18) | 98 (44) |
| **TDI (24 months)** |  |  |  |
| N (Missing) | 40 (33) | 40 (29) | 80 (62) |
| **HAQ-DI (24 months)** |  |  |  |
| N (Missing) | 53 (20) | 53 (16) | 106 (36) |
|  | | | |
